# Prevalence of hepatitis B and C in blood transfusion center, Oujda Morocco (2013-2015)

**DOI:** 10.11604/pamj.2019.33.163.18688

**Published:** 2019-07-03

**Authors:** Samira Boubker, Nassiba Zerrouki, Zaina Sidqi, Maria Moussi, Amine El Mekkaoui, Wafaa Khannoussi, Ghizlane Kharrasse, Zahi Ismaili

**Affiliations:** 1 Hepato-Gastro-Enterology Unit Mohammed VI University Hospital Oujda, Oujda, Morocco; 2 Blood transfusion center, Oujda, Morocco Samira Boubker, Hepato-Gastro-Enterology Unit Mohammed VI, University Hospital Oujda, Oujda, Morocco

**Keywords:** Blood donors, developing country, hepatitis B, hepatitis C, Morocco prevalence

## Abstract

Viral hepatitis is a serious public health problem. Its epidemiology is not precisely known in Morocco. Our objective was to assess the prevalence of HBV and HCV in a particular population of “blood donors” at the Regional Blood Transfusion Centre in Oujda. A retrospective study was conducted from May 1, 2013 to May 31, 2015. Thirty-one thousand nine hundred and fifty-two blood donors were tested. Antigen detection was made according to ELISA technique (Monolisa^TM^HBs Ag ULTRA). HCV research was performed by ELISA using the kit « Monolisa HCV Ag-Ab ULTRA ». 177 blood donors included, they are divided into 155 male (87.6%) and 22 female (12.4%) subjects with a ratio of 7. The average age was 37.64 ± 12 years. Six cases were positive for HCV with an overall prevalence of 0.02%. The population study by sex shows a prevalence of 0.004% for 23177 male sera and 0.057% for 8775 female sera. Six donors were HCV positive, of which 05 were female (83.33%) and one was male (16.66%). The average age was 43 ± 14 years. Co-infection with HCV HBV-HCV and HCV-Syphilis and HCV-HIV are absent. Co-infection with HBV and HIV was found in one case. HBV-syphilis co-infection was found in 04 cases. Chronic viral hepatitis is a real global health problem. Its prevalence is currently estimated at 0.55% for HBV and 0.02% for HCV, reclassifying Morocco as a low endemic area. The prevention remains the most effective method to successfully control HBV and HCV infection.

## Introduction

Hepatitis Virus B (HBV) and C (HCV) Viruses are a major public health problem worldwide. The World Health Organization (WHO) has estimated that 2 billion people have been infected with HBV and that approximately 350-400 million of them are chronic carries of HBV surface antigen (HBs Ag) [1, 2]. One hundred seventy (170 million) chronic HCV cases or 3% of the general population with a large geographical variation, have high risk of progression to cirrhosis and hepato-cellular carcinoma (HCC) [3]. It is estimated that more than 300,000 new cases of HCC are observed annually [3]. One million people die each year from this disease because of the complications of chronic hepatitis and its consequences. The epidemiology of HBV and HCV infection in Morocco was studied. Through a recent Moroccan study on the general population, the WHO has estimated that 3 million of people are chronically infected with hepatitis B or C. This figure is an alarming sign and reclasses Morocco among the countries where these diseases are endemic in the world [2, 3]. Before the introduction of the hepatitis B vaccination into the immunization program, the WHO concluded that Morocco has an intermediate prevalence of hepatitis B. Currently, the epidemiology of hepatitis B is not precisely known in our country [1, 2]. The main objective of this study was to assess the prevalence of HBV and HCV in a particular population of “Blood Donors” at the Regional Blood Transfusion Center of Oujda.

## Methods

**Figure 1:**
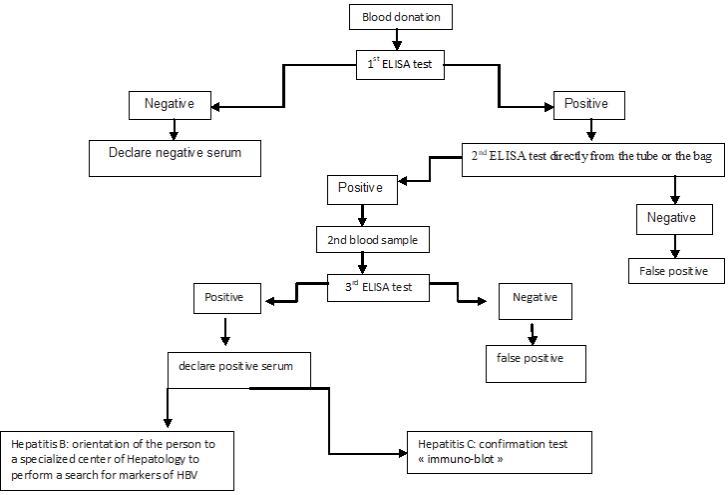
Age distribution of blood donors with HBs Ag (+) and HCV (+)diagnosis strategy of HBV and HCV infection and monitoring of seropositive person at the regional blood transfusion center of Oujda

This is a retrospective descriptive study conducted at the Regional Blood Transfusion Center of Oujda from 1^st^May 2013 to 31 May 2015. Our study is comprised of 31 952 blood donors including 23 117 males (72%) and 87,75 females (27%) with a male/female sex ratio of 2:6. Screening for HBs Ag was performed by enzyme immunoassay of ELISA (Enzyme Linked Immuno Sorbent Assay) using the kit «Monolisa^TM^HBs Ag ULTRA». HCV research was performed by ELISA using the kit «Monolisa HCV Ag-Ab ULTRA» for the qualitative detection of anti HCV Ab and capsid antigen in serum or plasma. The confirmation test was conducted by the kit «L'INNO-LIA^TM^ HCV Score». The dosage of ALT was performed using a spectrophotometer brand «Multiskan Ascent» preset on the wavelength λ= 340nm. The reagent used is Fluitest^®^ GPT ALT (Figure 1).

## Results

The prevalence of HBs Ag in blood donors was 0.55%. We included 177 blood donors who were divided into 155 male subjects (87,6%) and 22 female subjects (12,4%) with a sex ratio of 7 (Table 1). Their mean age was 37.64 ± 12 years with extremes of age from 18 to 64 years. Six cases were positive for HCV with a global prevalence of 0.02%. The study of the population by sex shows a prevalence of 0.004% for 23177 male serums and 0.057% for 8775 female serums. Six donors were positive for HCV including 05 donors were female (83.33%) and a donor was male (16.66%) with a female/male sex ratio of 5 (Table 2). The average age was 43 ± 14, 5 years with extremes of age from 25 to 60 years. The HBV-HCV co-infection and HCV-syphilis and HCV-HIV are absent. The HBV-HIV co-infection was found in one case. The HBV-syphilis co-infection was found in 04 cases. Among 183 infected serum (177 HBs Ag+ and 6 HCV+), 5 donors (2.7% of infected donors, 0.016% of donors in the study) had high levels of ALAT, 4 Donors had positive HBs Ag and a donor was HCV positive (Table 3).

**Table 1:** Sex distribution of blood donors with HBs Ag (+)

	Number of donations			Male HBs Ag (+)		Female HBs Ag (+)		Total HBs Ag (+)	
	Male	Female	Total	Number	Prevalence (%)	Number	Prevalence (%)	Number	Prevalence (%)
From May 2013 to April 2014	11092	4110	15202	65	0.59%	9	0.21%	74	0.8%
From May 2014 to May 2015	12085	4665	16750	90	0.74%	13	0.28%	103	1.02%
**Total**	23177	8775	31952	155	0.67%	22	0.25%	177	0.55%

**Table 2:** Sex distribution of blood donors with HCV (+)

	Number of donations			Male HCV (+)		Female HCV (+)		Total HCV (+)	
	Male	Female	Total	Number	Prevalence	Number	Prevalence	Number	Prevalence
From May 2013 to April 2014	11092	4110	15202	1	0.01%	3	0.07%	4	0.026%
From May 2014 to May 2015	12085	4665	16750	0	0.00%	2	0.04%	2	0.012%
**Total**	23177	8775	31952	1	0.004%	5	0.057%	6	0.02%

**Table 3:** Age distribution of blood donors with HBs Ag (+) and HCV (+)

	18-29 years		30-45 years		> 45 years	
	HBV	HCV	HBV	HCV	HBV	HCV
From May 2013 to April 2014	20	2	31	0	8	3
From May 2014 to May 2015	46	0	65	1	7	0
Total	66	2	96	1	15	3
Percentage (%)	37.28	33.33	54.23	16.66	8.5	50

## Discussion

### Screening tests

**Hepatitis B:** HBs Ag is the principal marker used in blood donation screening programs. The presence of HBs Ag may indicate current or chronic infection and potential infectivity. Anti-HBc antibody was subsequently produced in acute infection, after the appearance of HBs Ag and marks the beginning of the immune response to infection with HBV; this antibody remains present throughout life. The presence of anti-HBs playing a protective role, it would be necessary to seek anti-HBs in all reagents donations for anti-HBc to distinguish between contagious individuals and not contagious. Negative donations for HBs Ag, reactive for anti-HBc and anti-HBs having a concentration of 100 mIU/mL or greater are generally considered safe and acceptable for release for clinical use or the manufacture of other products. Systematic detection of HBV DNA in blood donors is not recommended by the WHO. In Morocco, only HBs Ag is tested for hepatitis B among blood donors, it is the same for the Maghreb countries including Tunisia and Algeria [4]. In France, the detection of the DNA of the hepatitis B virus, in addition the HBs Ag, has been justified by the reduction even more risk of transmission of this virus by transfusion of infected donations during the acute phase of the infection [5]. In our study, the surface antigen (HBs Ag) is the serological marker used in screening for HBV, the test used is the immunoassay test of ELISA (Enzyme Linked Immuno Sorbent Assay) using the kit “MONOLISA HBs Ag ULTRA ” [4, 6-10].

**Hepatitis C:** the HCV identification uses the following targets: Serological markers: anti-HCV antibodies and HCV Ag; the viral nucleic acid: HCV RNA. In Morocco, only serology for hepatitis C is mandatory in blood donors [3]. In France, in addition the serology, the research for HCV RNA is obligatory for any blood donations [5]. In our study, hepatitis C was identified by the research of antigen and hepatitis C antibodies. Kit “Monolisa HCV Ag- Ab ULTRA ”has been used for screening and Kit“ INNO -LIA HCV ” for confirmation of HCV [5, 9].

### Sample: the population of blood donors

In our study, there was a significant increase in the number of blood donors at the Regional Blood Transfusion Center of Oujda compared to the number of donors in 1999 (6440 donors) and 2000 (8075 donors) [11]. The blood donation rate reached about 2.76% of Oujda's estimated population of 551 767 in 2014, which is in line with WHO targets (2%). Our results do not match the data of a study on “the epidemiological surveillance of blood donors in France between 1992 and 2002” which aimed to reduce the number of blood donors who spent more than 6% of the general population in 1992 to 4% in 2002 [5]. This decrease is explained by the decrease in the need for blood products due to the revision of transfusion indications, the development of endoscopic procedures and autologous transfusion and preventive measures for donor selection. In our study, there was a predominance of male donors (72%), the number of female donors tripled in 2000 [11].

### The prevalence of HBs antigen

The global distribution of hepatitis B is highly variable, defining three geographical categories according to the prevalence of Hbs Ag [12-14]: i) Highly endemic areas correspond to a HBs Ag prevalence of more than 8% in sub-Saharan Africa, Southeast Asia and the Far East; ii) Intermediate endemic areas where the prevalence of HBs Ag is between 2 and 8%, they cover the Mediterranean, Eastern Europe and Latin America; iii) Low endemic areas where HBs Ag prevalence is less than 2% are mainly represented by Western Europe, North America and Japan. In our study, the results of HBs Ag screening show a prevalence of 0.55%, which ranks Morocco among the low endemic countries. However, according to WHO data, and before the introduction of the hepatitis B vaccine into the vaccination program, Morocco was considered to have an intermediate prevalence of hepatitis B [1, 2]. By comparing the values of our study with those of other countries, we find that our figures are higher than those observed in France (0.065%) [15]. However, the prevalence of HBs Ag in our series is lower than that observed in Tunisia (3.6%) [16] and Mauritania (20.3%) [17]. There was also a significant decrease in the prevalence of HBs Ag among blood donors at the Regional Blood Transfusion Centre compared of Oujda to a study conducted in the same centre by Dr. MOUSSI between 1996 and 2000, involving 28,941 blood donors, the target prevalence of HBs Ag of 1.8% [11]. This decrease in hepatitis B prevalence is due to the success of the national HBV vaccination program. In our study, the prevalence of HBs Ag was significantly higher among people aged 30 to 45 years (54.23% of HBs Ag + donors), followed by people aged 18 to 29 years (37.28%). This is because this age corresponds to a period of unprotected sexual activity, without tattoos and organ piercing. Our results are consistent with those obtained in Dr. MOUSSI's study [11].

### HCV prevalence

The WHO estimated that in 1999, 2.9% of the world's population was living with HCV infection [18], knowing that no data were available in 57 countries. In Morocco, the exact prevalence of HCV infection is not well known. The WHO classifies Morocco in an area of average HCV prevalence, with a prevalence ranging from 1 to 2.49% [19]. In our study, the prevalence of HCV infection is 0.02%, which places the Oujda region in the low prevalence area, these results show a significant decrease in HCV prevalence compared to Dr Moussi´s study conducted at the same centre between 1996 and 2000 which indicated a prevalence of 0.42% [11], we also note a low prevalence compared to studies on blood donors in different transfusion centers in Morocco with a maximum prevalence (1.1%) in Casablanca [19]. Compared to the Maghreb countries, the prevalence of HCV positive serum in our series is lower than that observed in Tunisia (0.56%) [20], Algeria (0.2%) [21] and Mauritania (1.1%) [17]. The figures in our series are in addition to those recorded in France with a prevalence of 0.03%, in an interesting study of 383,000 blood donors in 2012 [5]. In Africa, there is a very high heterogeneity of prevalence rates observed, ranging from 0% in Zambia to 26.6% in Egypt [22]. In our study, we found a higher prevalence of anti-HCV among female blood donors (83.33%), this predominance of women can be explained by some of the more common social risk practices among women (blood transfusion during delivery, tattooing). There is also a prevalence of 50% in the > 45 age group, which is also found in Dr. Moussi's study at the Regional Blood Transfusion Centre in Oujda (1996-2000) [11].

### Dosing of ALT

When the hepatitis C screening test is introduced, the search for increased ALT levels is not identifiable in terms of improving transfusion safety according to the WHO and for this reason, routine ALT testing of blood donors is not recommended [8]. In Morocco, the systematic determination of ALT in blood donors is obligatory [23]. In France, the introduction of viral genomic screening of blood donors has cancelled the contribution of the ALT test to blood safety in relation to the known hepatotropic virus. The determination of ALT in France on blood donations was abolished in 2003 [5]. In our study, among 183 infected donors (HBV and HCV), 05 donors (0.016% of the study donors, 2.7% of the infected donors) had high levels of ALT, 04 HBs Ag and one hepatitis C positive donor. This low prevalence of high ALT levels in infected subjects requires the interest and value of the ALT test for transfusion safety.

### Co-infection

In Dr MOUSSI's study conducted at the Regional Blood Transfusion Centre in Oujda (1996-2000), no cases of HBs Ag (+)/HIV (+)/HIV (+) co-infection were reported. In Congo, a study was carried out by F. BALEKA at the Blood Transfusion Centre in Kinshasa between October and December 2000 on 373 blood donors and its objective is to clarify the co-infection that exists between the 3 viruses (HBV, HCV and HIV). 2 cases of HBs Ag /HIV co-infection were reported, representing a prevalence of 0.005% [24]. In our study, no cases of HCV/HIV co-infection were noted. This can be explained by the low number of HCV-positive donors. Our results are consistent with the results observed in the F. BALEKA study in Congo and in Dr. Moussi's study at the regional blood transfusion centre in Oujda. In our study, no cases of HBV/HCV co-infection were noted. This can be explained by the low prevalence of HCV in our series. In Morocco, the study found 0.13% co-infection with HBV and HCV [25], compared to 0.03% co-infection in the study by Zouhdi *et al.* [26]. In our study, 4 cases are Hbs Ag+ /VDRL+ or a prevalence of 0.0001% and there were no HCV+ /VDRL+ cases. Benkirane *et al*. found 0.33% of cases that are both Hbs Ag+ /VDRL+ and 0.022% of cases that are both HCV+ /VDRL+ [26]. Zouhdi *et al*. found 0.05% of Hbs Ag+ /VDRL+ cases [26]. In Dr. Moussi’s study, 2 cases were Hbs Ag+/VDRL+ and no HCV+/VDRL+cases.

## Conclusion

Chronic viral hepatitis is a real global health problem. In Morocco, the epidemiology of hepatitis B (HBV) and C (HCV) is not precisely known. This study, which involves a large number of blood donors (31 952), assessed the local prevalence of HBV and HCV in this population. According to this study, this with the available therapies, it is possible to achieve in the majority of patients a cure for HCV or prolonged disease control for HBV. The overall strategy for controlling hepatitis B is to control the disease. There are highly immunogenic vaccines available to prevent infections caused by hepatitis B, these infections are the first global diseases that can be prevented by vaccination in terms of morbidity and mortality. Viral hepatitis has been declared a public health priority by the WHO, along with HIV, tuberculosis and malaria. In Morocco, the introduction of the hepatitis B vaccine into the national immunization schedule has been obligatory for all newborns since 1999. That is a major step forward for our country. The HBV vaccine is the first vaccine against a sexually transmitted infection and can be considered the first vaccine against cancer. Ultimately, only screening and education, particularly among high-risk groups, will prevent this serious disease.

### What is known about this topic

- Chronic viral hepatitis is a real global health problem;
- In Morocco, the epidemiology of hepatitis B (HBV) and C (HCV) viruses is not precisely known;
- 0,64The World Health Organization (WHO) has estimated that 2 billion people have been infected with HBV.

### What this study adds

- Our study assesses the prevalence of HBV and HCV in a particular population of “blood donors” at the Regional Blood Transfusion Centre in Oujda;
- In our study, the prevalence is currently estimated at 0.55% for HBV and 0.02% for HCV, which places Morocco among the low endemic countries;
- The prevention remains the most effective method to successfully control HBV and HCV infection.

## Competing interests

The authors declare no competing interests.
